# Risk factors for stillbirth and early neonatal death: a case-control study in tertiary hospitals in Addis Ababa, Ethiopia

**DOI:** 10.1186/s12884-021-04025-8

**Published:** 2021-09-21

**Authors:** Eskinder Kebede, Melani Kekulawala

**Affiliations:** 1 Department of Obstetrics and Gynecology, College of Health Sciences, Addis Ababa University, Addis Ababa, Ethiopia; 2 Department of Health Behavior and Health Education, University of Michigan, Ann Arbor, USA; 3 College of Medicine and Life Sciences, University of Toledo, Toledo, USA

**Keywords:** Stillbirth, Early neonatal death, Perinatal mortality

## Abstract

**Background:**

Ethiopia is a Sub-Saharan country that has made significant improvements in maternal mortality and under-five mortality over the past 15 years. However, the nation continues to have one of the highest rates of perinatal mortality in the entire world with current estimates at 33 deaths per 1000 live births.

**Methods:**

This case-control study was conducted between October 2016 and May 2017 at Tikur Anbessa Hospital and Gandhi Memorial Hospital. All women who had a stillbirth or early neonatal death (i.e. death within 7 days) during this period willing to participate were included as cases. A systematic random sample of women delivering at the hospital were approached for recruitment as controls to generate a 2:1 ratio of controls to cases. Data on risk factors were retrieved from medical records including delivery records, and treatment charts. Statistical differences in background and social characteristics of cases and controls were determined by t-test and chi-squared (or fisher’s exact test) for quantitative and categorical variables respectively. Binary logistic regression analysis was completed to determine any associations between risk factors and stillbirth/early neonatal death.

**Results:**

During the study period, 366 women delivering at the hospitals were enrolled as cases and 711 women delivering at the hospitals were enrolled as controls. Records from both hospitals indicated that the estimated stillbirth and neonatal mortality rates were 30.7 per 1000. Neonatal causes (43.4%) were the most common, followed by antepartum (32.5%) and intrapartum (24.5%). Risk factors for stillbirths and early neonatal death were low maternal education (aOR 1.747, 95%CI 1.098–2.780), previous stillbirth (aOR 9.447, 95%CI 6.245–14.289), previous preterm birth (aOR 3.620, 95%CI 2.363–5.546), and previous child with congenital abnormality (aOR 2.190, 95% 1.228–3.905), and antepartum hemorrhage during pregnancy (aOR 3.273, 95% 1.523–7.031).

**Conclusion:**

Antepartum hemorrhaging is the only risk factor in our study amenable for direct intervention. Efforts should be maximized to improve patient education and antenatal and obstetric services. Moreover, the most significant cause of mortality was asphyxia-related causes. It is imperative that obstetric capacity in rehabilitation services are strengthened and for further studies to investigate the high burden of asphyxia at these tertiary hospitals to better tailor interventions.

## Background

With more than 5 million perinatal mortality occurring worldwide annually, ending preventable perinatal mortality by 2030 is an essential target of the third Sustainable Development Goal [1]. Perinatal mortality is defined by the World Health Organization as the number of fetal deaths past 28 (or 22) completed weeks of pregnancy (i.e. stillbirth) plus the number of deaths among live-born children up to 7 completed days of life (i.e. early neonatal death) [2]. The rate of mortality during the perinatal period is higher than in any other period of an individual’s life. Moreover, perinatal mortality is a significant indicator of the health status of a nation as it reflects maternal conditions during pregnancy, intrapartum conditions, and quality of delivery care.

More than 95% of perinatal mortality occur in developing countries with South Asia and Sub-Saharan Africa (SSA) bearing the largest burden [3]. The perinatal mortality rate of Sub-Saharan Africa is estimated to be nearly 34.7 per 1000 births. Ethiopia is a Sub-Saharan country that made significant improvements in maternal mortality and under-five mortality over the past 15 years. However, perinatal mortality continues to be a major obstacle and the country continues to have one of the highest rates of perinatal mortality in the entire world with current estimates at 33 deaths per 1000 live births [4]. Despite offering universal obstetric services, including prenatal care, the nation has faced systematic obstacles such as shortages and inadequacies in staff, ambulatory transportation, and equipment. Rural and cultural stigma against utilizing certain maternal health services also serve as obstacles. Moreover, some facilities in Ethiopia continue to charge user fees for maternal health services and such facilities can offer better services and amenities [5]. Thus, despite sustained efforts to address these issues, several obstacles continue to prevent declines in stillbirth rates.

There is limited data on perinatal mortality and risk factors of perinatal mortality in Ethiopia. The majority of research efforts are at the national level and thus, there is minimal data in literature on local contexts. It is critical to examine local contexts as well since national data may overlook important regional or local patterns. There are however a few studies focused on determinants of perinatal mortality in local contexts. A perinatal mortality audit at Jimma hospital during the decade of 1990 found that the majority of perinatal mortality were due to mechanical factors related to the peripartum periods [6]. The study identified obstructed labor with and without ruptured uterus to be the single most significant cause of perinatal mortality and attributed to 37.4% of cases. Other cases identified were hypertensive disorders (6.7%), lethal congenital anomalies (1.4%), antepartum hemorrhage (2.1%), prematurity (7.2%) and unknown (28.9%). A prospective longitudinal study was conducted in northwest Ethiopia and found that previous stillbirth, twin birth, not receiving tetanus toxoid vaccine during pregnancy, short birth interval of less than 24 months, maternal illiteracy, and mother running own business were significant predictors of perinatal mortality [7]. An unmatched case-control study at three hospitals of Addis Ababa, Ethiopia found that birth weight, lack of prenatal care, and history of past perinatal loss were significant factors for perinatal mortality [8]. A recent retrospective cross-sectional study examined the relationship between socioeconomic and demographic variables and perinatal mortality in the Tigray region of Ethiopia [9]. Overall, the prevalence of experiencing perinatal mortality was 6.2% in the Tigray region and the study identified sex of child, previous birth intervals, availability of waste facilities, wealth index, birth type, mother’s age, parity, place of residence, mother’s occupation and source of drinking water as factors significantly associated with perinatal mortality. Lastly, a case-control study in Southwest Ethiopia found that being primipara, previous history of perinatal mortality, and obstetric complication during labor significantly increased perinatal mortality [10]. Thus, while there is some literature on perinatal mortality rates and risk factors of perinatal mortality, a far greater amount is necessary in order to formulate interventions and policies to improve the current situation of maternal and child health.

This study strives to help fill the gaps in literature on risk factors of perinatal mortality at a local context. Moreover, the study specifically utilizes two large tertiary referral hospitals. The outcomes of this study can be utilized at both regional and national levels to help influence policy and future health interventions. This study can also serve to help other researchers who further analyze these topics.

## Methods

### Study design

This study was an unmatched case-control design conducted between October 2016 and May 2017 at Tikur Anbessa Hospital (TAH) and Gandhi Memorial Hospital (GMH). For this study, women with live births during the study period who were willing to participate were randomly selected as controls. Women with antepartum, intrapartum, and early neonatal death (i.e. death within 7 days) willing to participate were selected as cases. Neonates who were born at the hospital but died at home after discharge were excluded from this study. This was due to difficulties with follow-up as many patients are transported from regional and district hospitals and do not seek continuity of care at these tertiary hospitals. Neonates delivered outside the hospitals and referred to the neonatology ward at the hospital were excluded from this study. A total of 1077 participants were recruited during the study period: 711 controls and 366 cases for approximately a 2:1 ratio for controls to cases.

### Study setting

The study was conducted in the labor wards of two government-funded tertiary hospitals: Tikur Anbessa Hospital (TAH) and Gandhi Memorial Hospital (GMH). Both hospitals serve as teaching hospitals that provide a range of obstetric and gynecological services and accept a large number of referrals from Addis Ababa and the surrounding districts. Thus, both hospitals experience a higher rate of delivery than those in their surrounding areas. Women with high-risk pregnancies may be admitted to the maternity ward before labor for planned delivery. After delivery, neonates who need special care are transferred to the neonatology wards at the Neonatal Intensive Care Unit.

### Participants

All women with stillbirth or early neonatal death occurring at Tikur Anbessa Hospital and Gandhi Memorial Hospital during the study period willing to participate were included as cases. For every woman with stillbirth or early neonatal death, two randomly selected women with live births willing to participate were included as controls. Controls were selected from the general patient population at each hospital immediately after delivery. If for some reason a patient initially selected as a control then experienced early neonatal death, it was recategorized as a case.

### Data collection

For data collection, a recruiting team composed of midwives was created under the guidance of the principal investigator. Nine midwives from the labor ward in TAH and GMH were trained as recruiters and data collectors for the study. At least one of these recruiters were assigned to each shift at their respective hospitals. The entire obstetrics and gynecology staff at both hospitals were given presentations on the study at morning meetings. Recruiters were introduced to the staffs as leaders of recruitment. The majority of deliveries at the tertiary hospitals are conducted by resident OBGYNs with assistance of nurses and midwives. If a stillbirth or early neonatal death occurred, the recruiter on that shift was consulted. Mothers with stillbirth and early neonatal death were given a mourning period followed by a counseling session. After a careful mental, emotional, and physical stability assessment, a recruiter would approach the mother for the interest and consent of the study. Additionally, the recruiters randomly selected women with live births for the control population and approached them for consent after delivery before discharge. Study controls were selected by a random systematic sampling technique from a list of women who delivered at the hospitals each day The recruiter was given an envelope with randomly generated allocations from a staff member. Following a delivery, the allocation from the envelope would determine if that mother would be approached for recruitment. Recruitment occurred during business hours of the hospital. Once randomly selected, the control patients were approached by the recruiter. Data for the study were collected using a pre-designed data collection form by trained nurse assistants.

For both case and control participants, data on potential risk factors were retrieved from medical records including health passports, delivery records, and treatment charts shortly before discharge. The midwives asked for clarification of any incomplete data from the health care worker in charge of the case or the mother herself. Questions were asked of mothers only after they had proper time to mourn, received counseling, and were in a stable emotional, mental, and physical state. Specifically, interview questions regarding the maternal demographics, exposures, and prior obstetric history were asked from the mother. Interview questions regarding delivery, complications, and cause of death for cases were obtained from the physicians and other midwives who were involved in the care of that patient.

### Study instruments

Data were collected utilizing a data capture sheet composed of three sections. Section 1 captured data on demographic information, prior obstetric history, antenatal care, and complications of current pregnancy and labor. Section 2 captured data on stillbirth and early neonatal cases. All available information from laboratory tests, other investigations, and autopsies are also recorded in this section. If the cause of mortality was unknown, an autopsy was requested only if consent was received from the mother. The purpose of section 2 was to utilize all available information to assign a standard primary cause of mortality to each case based on the Wigglesworth Classification. Section 3 captured data on participants with multiple pregnancies and/or deliveries.

A verbal autopsy instrument modified from the World Health Organization (WHO) instrument for the evaluation of stillbirth and early neonatal death was used [11, 12]. Modifications included adjustments for cultural sensitivity and exclusion of irrelevant questions. The final questionnaire was composed of different sections for basic information about the deceased neonate and stillbirth and included both narrative and close-ended questions. The instrument was translated into Amharic language and then re-translated in English to ensure content recording and validity. Pretesting of the instrument was performed to identify potential issues during instrument administration and to drive possible solutions. A senior midwife conducted a verbal autopsy at the hospital. The health care provider who attended the birth did not participate in the interview for the verbal autopsy.

### Study variable definitions

The following variable definitions were utilized in this study:

- *Live birth*: the complete expulsion or extraction from the mother of a product of human conception, irrespective of duration of pregnancy, which shows any evidence of life (ie. Heartbeats, umbilical cord pulsations, breathing, or voluntary muscle movement), regardless of whether the umbilical cord has been cut or the placenta is attached
- *Stillbirth*: a baby born with no signs of life at or after 28 weeks of gestation
- *Antepartum Stillbirth*: the death of the fetus before initiation of labor after 28 weeks of gestation as diagnosed by the attending physician
- *Intrapartum Stillbirth*: the death of the fetus after initiation of labor after 28 weeks of gestation as diagnosed by the attending physician
- *Early neonatal death*: A neonate that born in the hospital but dies before the seventh postnatal day
- *Perinatal Mortality*: the sum of infant deaths that occur at less than 7 days of age and fetal deaths with a gestational age of 28 weeks or more

### Ethical clearance

Ethical clearance was obtained from the Research and Publication Committee (RPC) of the Department of Gynecology and Obstetrics and Institutional Review Board of the College of Health Sciences, Addis Ababa University (Record# 008/2016). Permission was also obtained from the study facilities to collect data. Participation in the study was completely voluntary and informed consent was acquired from every participant before participation. While this study involved women and newborns which are considered vulnerable populations, several efforts were made in the study design to protect their emotional and mental well-being regarding this sensitive topic. Anonymity and confidentiality of patient personal information were protected through several mechanisms.

### Classification of stillbirth and early neonatal death

Stillbirth and early neonatal death was classified according to the method described by Wigglesworth et al., which was utilized to identify a single underlying cause of mortality [13, 14]. While in most cases, there are several contributing factors of mortality, the Wigglesworth Extended Classification places events that occurred first in the hierarchy for the cause of mortality than those occurring later. Available health records and verbal autopsies of cases were reviewed separately by two midwife data collectors. Each was assigned a primary cause of mortality based on the Wigglesworth classification system. If there was a consensus between the two midwife-data collectors, the cause of mortality was assigned as such. However, if there was a discrepancy, the principal investigator was consulted and reviewed the case to assign the final cause of mortality. Initially, the study had intended to categorize stillbirths as fresh or macerated; however, much of the data on this was unavailable or undocumented by the midwife recruiters so it was therefore left out of the analysis.

### Statistical analysis

IBM SPSS Statistics Version 26 was utilized for statistical analysis. A comparison of demographic and obstetric characteristics for the case and control population was done using Pearson’s chi-squared and fisher’s exact test for categorical variables. Comparisons of the mean for quantitative variables between the two populations were done using a t-test with equal variance not assumed. Univariate logistic regression analysis was performed to test the association between variables and stillbirth. For those variables that showed a significant association in the univariate analysis, multivariate logistic regression was created to adjust for confounders. The variables investigated in the multivariate model were education, marital status, blood type, parity, previous stillbirth, previous preterm birth, previous child with congenital abnormalities, antenatal care for current pregnancy, hypertensive disorder during pregnancy, antepartum hemorrhage during pregnancy, PROM during pregnancy, method of delivery, and multiple births. Blood type was included as a variable because some studies have demonstrated an association between blood type and bleeding status, specifically postpartum hemorrhage [15, 16]. A data replacement method for missing variables was not utilized because missing data composed less than 5% per variable.

## Results

During the study period, there were 11,916 deliveries total in both tertiary hospitals. A total of 1077 women were enrolled in the study; 711 women were enrolled as controls and 366 were enrolled as cases. Demographic and social characteristics of both the case and control populations were compared (Table 1). The mean maternal age for the control and case populations was 26.80 and 27.23, respectively. The demographic characteristics of age, religion, residence, and occupation were not statistically significantly different between cases and controls. Cases were sub-classified as antepartum, intrapartum, and early neonatal death (Table 2).

**Table 1:** Social and demographic characteristics of cases and controls

Variable	Controls *N* = 711	Cases *N* = 366	*p*-value
Maternal Age in Years	26.80 +/− 4.42	27.23 +/− 4.87	0.06
Religion			0.56
Orthodox	480 (67.5)	244 (66.7)	
Catholic	15 (2.1)	8 (2.2)	
Protestant	54 (7.6)	27 (7.4)	
Muslim	161 (22.6)	83 (22.7)	
Other	1 (0.0)	4 (1.0)	
Residence			0.42
Urban	629 (88.5)	327 (89.3)	
Rural	82 (11.5)	39 (10.7)	
Occupation			0.06
Housewife	391 (55.0)	201 (54.9)	
government-employed	123 (17.3)	59 (16.1)	
Private-Employed	132 (18.6)	70 (19.1)	
Merchant	56 (7.9)	26 (7.1)	
Student	6 (0.8)	5 (1.4)	
Other	3 (0.4)	5 (1.4)

**Table 2:** Cases Sub-classification based on time of mortality

Case Classification	Total, n (%)
Antepartum	119 (32.5)
Intrapartum	75 (24.5)
Early neonatal	159 (43.4)
Unknown	13 (3.6)

Based on verbal autopsy and available health records, the cause of mortality attributed to each case was classified via the Wigglesworth classification (Table 3). The highest cause of mortality was related to intrapartum asphyxia, anoxia, or trauma, followed by congenital anomalies, umbilical cord-related complications, and preeclampsia, respectively.

**Table 3:** Causes of Stillbirth at Tikur Anbessa Hospital and Gandhi Memorial Hospital

Cause of Mortality	Total N = 366 n (%)
Congenital Anomaly	59 (16.1)
Unexplained Antepartum Fetal Mortality	38 (10.4)
Mortality from Intrapartum Asphyxia, Anoxia, Trauma	148 (40.4)
Immaturity/ Prematurity	33 (9.0)
Sepsis	10 (2.7)
Other Fetal-Related Causes	7 (1.9)
Cord-Related Cause	28 (7.7)
Placenta-Related Cause	3 (0.8)
Uterus-Related Cause	4 (1.1)
Preeclampsia/Eclampsia	24 (6.6)
Unclassified	12 (3.3)

Univariate logistic regression analysis demonstrated that the following risk factors were significant predictors of stillbirth or early neonatal death: lower maternal education, marital status, blood type, parity, previous still birth, previous preterm birth, ever had child with congenital abnormalities, antenatal care for current pregnancy, hypertensive disorder during pregnancy, antepartum hemorrhage in pregnancy, PROM during pregnancy, method of delivery, multiple birth (Table 4). Multivariate logistic regression was conducted to adjust for interactions of variables (Table 5). In this analysis, risk of stillbirth and early neonatal death was still increased among women with lower maternal education, parity, previous stillbirth, previous preterm birth, previous child with congenital abnormality, and antepartum hemorrhage during pregnancy.

**Table 4:** Univariate logistic regression analysis for the likelihood of Stillbirth

Variable	Crude OR	95% CI	p-value
Education			0.01
No Education	1.72	1.03–2.87	
Some Education (Primary-Secondary)	1.82	1.26–2.62	
Higher Education/Vocational	Ref		
Marriage Status			
Currently Married	Ref		
Not Currently Married	2.19	1.20–3.98	0.01
Employment			
Employed	Ref		
Unemployed	1.06	0.82–1.37	0.65
Age			
< 35	Ref		
≥ 35	1.23	0.78–1.93	0.15
Income Quintile			0.20
Poorest	1.11	0.72–1.71	
Poorer	1.25	0.83–1.89	
Middle	0.78	0.51–1.20	
Rich	0.82	0.52–1.31	
Richest	Ref		
BMI			0.53
Normal	Ref		
Low	1.28	0.53–3.03	
High	0.87	0.64–1.18	
Blood Type			0.04
Type A	1.41	1.03–1.92	
Type B	0.89	0.63–1.26	
Type AB	1.42	0.85–2.38	
Type O	Ref		
Parity			< 0.01
0	Ref		
1–5	0.24	0.06–0.96	
6 or more	1.83	0.28–12.07	
Previous Still Birth			
No	Ref		
Yes	10.02	7.01–14.34	< 0.01
Previous Preterm Birth			
No	Ref		
Yes	4.59	3.32–6.34	< 0.01
Ever Had Child with Congenital Abnormalities			
No	Ref		
Yes	2.44	1.58–3.77	< 0.01
Antenatal Care for Current Pregnancy			0.02
No ANC	1.96	0.85–4.48	
1–2 visits	1.79	1.09–2.93	
3 or more visits	Ref		
HIV			
No	Ref		
Yes	1.76	0.78–3.97	0.17
Hypertensive Disorder during Pregnancy			
No	Ref		
Yes	1.73	1.26–2.37	< 0.01
Antepartum Hemorrhage in Pregnancy			
No	Ref		
Yes	4.76	2.45–9.07	< 0.01
Intrauterine Growth Restriction in Pregnancy			
No	Ref		
Yes	1.66	0.86–3.19	0.13
PROM during Pregnancy			
No	Ref		
Yes	0.62	0.40–0.95	0.03
Other Complications in Pregnancy			
No	Ref		
Yes	1.88	0.85–4.17	0.12
Method of Delivery			0.02
SVD	Ref		
Cesarean section	0.81	0.56–1.11	
Other	0.36	0.17–0.77	
Multiple Birth			
No	Ref		
Yes	2.70	1.66–4.38	< 0.01
Sex of Baby			
Male	0.95	0.73–1.23	0.68
Female	Ref

**Table 5:** Multivariate logistic regression analysis for the likelihood of Stillbirth

Variable	Adjust OR	95% CI	p-value
Education			0.05
No Education	1.29		
Some Education (Primary-Secondary)	1.75	0.66–2.56	0.45
Higher Education/Vocational	Ref	1.10–2.78	0.02
Marriage Status			0.39
Currently Married	Ref		
Not Currently Married	1.42	0.64–3.12	
Blood Type			0.18
Type A	1.38	0.93–2.04	
Type B	0.93	0.60–1.44	
Type AB	1.57	0.83–2.96	
Type O	Ref		
Parity			0.01
0–1	Ref		
2–5	0.14	0.01–1.90	
6 or more	1.11	0.06–2.48	
Previous Still Birth			< 0.01
No	Ref		
Yes	9.45	6.26–14.29	
Previous Preterm Birth			< 0.01
No	Ref		
Yes	3.62	2.36–5.55	
Ever Had Child withCongenital Abnormalities			0.01
No	Ref		
Yes	2.19	1.23–3.91	
Antenatal Care for Current Pregnancy			0.90
No ANC	Ref		
1–2 visits	1.301	0.43–3.96	
3 or more visits	0.99	0.52–1.89	
Hypertensive Disorder during Pregnancy			0.61
No	Ref		
Yes	1.12	0.73–1.71	
Antepartum Hemorrhage in Pregnancy			< 0.01
No	Ref		
Yes	3.27	1.52–7.03	
PROM during Pregnancy			0.56
No	Ref		
Yes	0.85	0.50–1.45	
Method of Delivery			0.61
SVD	Ref		
Cesarean section	0.86	0.58–1.28	
Other	0.71	0.29–1.73	
Multiple Birth			0.08
No	Ref		
Yes	1.77	0.95–3.31	

## Discussion

During our study period of October 2016 to May 2017, the overall perinatal mortality rate was 30.1 per 1000 live births between both tertiary hospitals. According to the EDHS report, the overall national stillbirth and early neonatal death rate was 46 per 1000 live births [1]. Our study rate is lower than the WHO estimate of 58 per 1000 births in East African countries [2]. This rate is also lower than the rates of stillbirth and early neonatal death in Southern Ethiopia and Nigeria, which were 85 per 1000 live births and 53.9 per 1000 live births, respectively [17, 18]. This discrepancy could be due to the fact that both Tikur Anbessa and Gandhi Memorial hospitals are located in Addis Ababa, the nation’s capital. Thus, while patients transferred to a tertiary referral center are often more severely ill, there are more physicians, technologies, transportation, and amenities located in Addis Ababa than any other region of the country. Also, the local population of Addis Ababa who utilize the services of GMH and TAH are high in socio-economic status that populations of the surrounding, rural regions. Moreover, the study was unable to include early neonatal death that did not occur at the hospital. Therefore, our documented study perinatal mortality may be falsely low for that reason.

Regarding demographic characteristics, we found that the risk for stillbirth and early neonatal death was higher among women who had lower maternal education and higher parity in multivariate analysis. Of note, the risk is only marginal for maternal education (p,0.048). High parity and low socio-economic status associations to stillbirth and early neonatal death have been reported by several other studies from developing countries, including Ghana and Brazil [19, 20]. We found obstetric factors to have the highest odds ratio associations with stillbirth and early neonatal death. This included previous stillbirth, previous preterm birth, and antepartum hemorrhage during pregnancy, and a previous child with congenital abnormalities. Studies done in Ethiopia and Zambia have similarly shown that prior preterm birth was an important risk factor associated with stillbirth and early neonatal death [21, 22]. We found that congenital anomalies accounted for 16.1% of stillbirth and early neonatal death in our study. This is similar to other reports that congenital anomalies account for 2.1–33.3% of stillbirths [23]. Similar results were reported in Nepal, where 18% of stillbirths were due to congenital anomalies in a study that examined medical records [24]. Finally, we found that antepartum hemorrhage during pregnancy was strongly associated as a cause of stillbirth in this study (aOR 3.273, 95% 1.523–7.031). A study involving 495 stillbirths in multiple West African countries showed a strong association between vaginal bleeding in late pregnancy and intrapartum bleeding with stillbirth outcomes [25].

Antepartum hemorrhage and preterm birth are the only risk factors amenable to direct intervention. A recent criteria-based audit for antenatal hemorrhage in a university hospital in eastern Ethiopia identified deficiencies in clinical documentation, clinical monitoring, limited resources for blood transfusion, as well as delays in management to be preliminary contributing factors [26]. Therefore, audits of the quality of care in GMH and TAH tertiary health facilities is essential to establish specific standards of care. Such standards might include a check-list criteria system for antepartum hemorrhage including vital sign monitoring, IV line set up, fluid administration, typing and crossing, and management of fluids. Moreover, emergency kits containing all essential supplies and relevant medications for emergent antepartum hemorrhage may also be a useful intervention for limited resource allocation. While interventions for preterm birth are still evolving, there is strong evidence suggesting that the use of low-dose aspirin as a potential intervention. In a large trail, daily low dose aspirin reduced the risk of preterm birth (before 37 weeks) by 11% and early preterm birth (before 34 weeks) by 25% among first time mothers [27]. Due to the low cost and safety of aspirin, it could easily be utilized and integrated into diverse settings, include low-resource settings globally.

While our study found antenatal care to be significant in univariate analysis, it was not a significant risk factor in multivariate analysis. While some studies in developing countries have found this to be a significant risk factor, others have not [28]. This may be due to expanded access to antenatal services that the country has prioritized in recent years or more likely due limitations of the study. Antenatal care was stratified into categories of none, 1–2 visits, and 3+ visits which may have affected its final statistical outcome. Moreover, the demographic characteristics of age, religion, residence, and occupation were not statistically significant in multivariate analysis, although several other studies have found demographic characteristics to be significant risk factors, particularly those of maternal age [1].

We also found that the majority (40.4%) of stillbirth and early neonatal death in our study were due to asphyxia, anoxia, or trauma. This is in contrast to a few previous studies done in Ethiopia that found that the majority of stillbirth and early neonatal death were primarily due to mechanical factors such as obstructed labor, uterine rupture, and malpresentation [17, 22, 26]. These previous studies found that nearly three-fourths of the cases were at term and the birth weight for more than half of the cases was in the normal range because the causative factor was mechanical-related. However, other studies have found that Sub-Saharan Africa contributes disproportionately to the global asphyxia mortality burden and accounts for 46% of morality [28, 29]. A regional survey documented that only 2–12% of labor room birth attendants have newborn resuscitation knowledge and only 8–22% of surveyed facilities had appropriate resuscitation equipment [28, 30]. However, there is an overall lack of understanding and investigation of asphyxia in these settings; often, even the definitive diagnosis of asphyxia is based on poor crying and low APGAR scores which have low predictive values [28, 31]. Lastly, functional multidisciplinary rehabilitative care teams are required for tertiary prevention of asphyxia. While developing countries have ample resources of this nature, SSA countries have rehabilitative care team worker densities below 0.01 [28, 32]. It may be useful for future researchers to investigate asphyxia as a cause of perinatal mortality in tertiary health centers in Ethiopia. Undoubtedly, strengthening obstetric capacity in rehabilitative service provision is essential. Moreover, further investigation on local asphyxia burden is imperative to create more specific and sustainable interventions.

There are several limitations to this study. The most significant limitation of this study is the exclusion of neonates who were born at the hospital but died at home after discharge. This was due to the inability to follow-up as many patients are transported from regional and district hospitals and do not seek continuity of care at these tertiary hospitals. Furthermore, causes of stillbirth versus causes of early neonatal death were not analyzed separately during this study and must be investigated in further studies to identify any differences. Moreover, the causes of stillbirth were assigned based on the clinical assessment of the providing physician. Fetal autopsies would have provided a more accurate cause of mortality data. Furthermore, there were deficiencies in methods of assessment for certain causes of mortality, including placental examination and genetic analyses. Additionally, due to financial and time constraints of the midwife recruiters and data collectors, the study was unable to continue for an entire year and an exact 2:1 ratio for controls to cases was unable to be met.

At with other case-control study designs, our study only demonstrates associations between risk factors and not necessarily causal relationships. Further research must be done to explore the causative relationship between such risk factors and perinatal mortality.

## Conclusion

Based on our study, efforts should be maximized to improve patient education, prenatal, antenatal, and obstetric services, particularly for women who have a history of obstetric complications identified as risk factors. Antepartum hemorrhaging is the only risk factor in our study amenable for direct intervention. Moreover, the most significant direct cause of mortality was asphyxia-related causes. Thus, efforts should be made to improve overall emergency obstetric care to improve pregnancy outcomes and to build obstetric capacity in rehabilitation services. Further studies are necessary to establish the causes of stillbirth and early neonatal death in this patient population. It is imperative that further studies investigate the high burden of asphyxia at these tertiary hospitals and SSA as a region. Lastly, assessments of psychological variables and their impact on subsequent pregnancy must also be considered in future studies.

## Data Availability

The datasets used and/or analyzed during the current study are available from the corresponding author upon reasonable request.

## Abbreviations

CI: Confidence Interval
GMH: Gandhi Memorial Hospital
SSA: Sub-Saharan Africa
TAH: Tikur Anbessa Hospital
WHO: World Health Organization

## References

[1] World Health Organization (WHO) (2018). Progress report: reaching every newborn national 2020 milestones.

[2] World Health Organization (WHO) (2016). The WHO application of ICD-10 to deaths during the perinatal period, ICD-PM.

[3] Akombi BJ, Renzaho AM (2019). Perinatal mortality in subSaharan Africa: a meta-analysis of demographic and health surveys. Ann Global Health.

[4] Central Statistical Agency (CSA) [Ethiopia] and ICF, The 2016 Ethiopia Demographic and Health Survey Key Findings, CSA and ICF, Addis Ababa, Ethiopia, and Rockville, Maryland, USA, https://dhsprogram.com/pubs/pdf/SR241/SR241.pdf. Accessed 6 Sept 2017.

[5] Pearson L, Gandhi M, Admasu K, Keyes EB (2011). User fees and maternity services in Ethiopia. Int J Gynecol Obstet.

[6] Gaym A. Perinatal mortality audit at Jimma hospital, South-Western Ethiopia, 1990-1999.Ethiopian Journal of Health Development. 2000;14(3):335–44. 10.4314/ejhd.v14i3.9907.

[7] Andargie G, Berhane Y, Worku A, Kebede Y. Predictors of perinatal mortality in rural population of Northwest Ethiopia: a prospective longitudinal study. BMC Public Health. 2013;13–168. 10.1186/1471-2458-13-168. 10.1186/1471-2458-13-168 PMC3599850 23433304

[8] Tilahun S, Gaym A (2008). Past reproductive performance and its correlation with perinatal mortality in the current gestation at teaching hospitals in Addis Ababa, Ethiopia. Ethiopian Med J.

[9] Woldeamanuel BT, Gelebo KK. Statistical analysis of socioeconomic and demographic correlates of perinatal mortality in Tigray region, Ethiopia: a cross-sectional study. BMC Public Health. 2019;19:1301. 10.1186/s12889-019-7642-z. 10.1186/s12889-019-7642-z PMC6796483 31619210

[10] Debelew GT (2020). Magnitude and determinants of perinatal mortality in Southwest Ethiopia. J Pregnancy.

[11] World Health Organization (2016). Verbal autopsy standards: the 2016 WHO verbal autopsy instrument.

[12] Aggarwal AK, Jain V, Kumar R (2010). Validity of verbal autopsy for ascertaining the causes of stillbirth. Bull World Health Organ.

[13] Wigglesworth JS. Monitoring perinatal mortality. A pathophysiological approach. Lancet. 1980 Sep 27;2(8196):684–6. 10.1016/s0140-6736(80)92717-8. 10.1016/s0140-6736(80)92717-8 6106794

[14] Hey EN, LLoyd DJ, Wigglesworth JS (1986). Classifying perinatal death: fetal and neonatal factors. Br J Obstet Gynaecol.

[15] Ali-Saleh M, Lavie O, Abramov Y. Evaluation of blood type as a potential risk factor for early postpartum hemorrhage. PLoS One. 2019 Apr 4;14(4):e0214840. 10.1371/journal.pone.0214840. 10.1371/journal.pone.0214840 PMC6448868 30947286

[16] Franchini M, Mengoli C, Lippi G (2016). Relationship between ABO blood group and pregnancy complications: a systematic literature analysis. Blood Transfus.

[17] Berhan Y, Berhan A. Perinatal mortality trends in Ethiopia. Ethiop J Health Sci. 2014;24(0 Suppl):29–40. 10.4314/ejhs.v24i0.4s. 10.4314/ejhs.v24i0.4s PMC4249204 25489181

[18] Ogunlesi TA, Ayeni VA, Ogunfowora OB, Jagun EO. The current pattern of facility-based perinatal and neonatal mortality in Sagamu, Nigeria. Afri Health Sci. 2019;19(4):3045–54. 10.4314/ahs.v19i4.26. 10.4314/ahs.v19i4.26 PMC7040347 32127880

[19] Aminu M, Unkels R, Mdegela M, Utz B, Adaji S, van den Broek N. Causes of and factors associated with stillbirth in low- and middle-income countries: a systematic literature review. BJOG. 2014;121(Suppl 4):141–53. 10.1111/1471-0528.12995. PMID: 25236649. 10.1111/1471-0528.12995 25236649

[20] Andrade LG, AM, Cunha A, Leite SR, Vital SA (2009). Fatores associados a ` natimortalidade em uma maternidade escola em Pernambuco: estudo Caso-controle (factors associated with stillbirth in a school maternity in Pernambuco: a case control study). Rev Bras Ginecol Obstet.

[21] Stringer EMVB, Killam WP, Giganti MJ, Mbewe R, Chi BH (2011). Determinants of stillbirth in Zambia. Obstet Gynecol.

[22] Hakim DBLY (2008). Stillbirth at Tikur Anbessa Hospital a retrospective study. Ethiopian J Reprod Health.

[23] McClure EM, SS, Pasha O, Goldenberg RL. Stillbirth in developing countries: a review of causes, risk factors, and prevention strategies. J Matern Fetal Neonatal Med. 2009;22(3):183–90. 10.1080/14767050802559129. 10.1080/14767050802559129 PMC3893926 19089779

[24] Vijayan VHJ (2012). Perinatal postmortem: factors influencing uptake and subsequent outcomes in an Asian population. Med J Malaysia.

[25] Chalumeau MB-CM, Breart G (2002). Can clinical risk factors for late stillbirth in West Africa be detected during antenatal care or only during labor?. Int J Epidemiol.

[26] Tura AK, Aboul-Ela Y, Fage SG, Ahmed SS, Scherjon S, Van Roosmalen J (2020). Introduction of criterion-based audit of postpartum hemorrhage in a university hospital in eastern ETHIOPIA: implementation and considerations. Int J Environ Res Public Health.

[27] Hoffman MK, Goudar SS, Kodkany BS, Metgud M, Somannavar M, Okitawutshu J, Lokangaka A, Tshefu A, Bose CL, Mwapule A, Mwenechanya M, Chomba E, Carlo WA, Chicuy J, Figueroa L, Garces A, Krebs NF, Jessani S, Zehra F, Saleem S, Goldenberg RL, Kurhe K, Das P, Patel A, Hibberd PL, Achieng E, Nyongesa P, Esamai F, Liechty EA, Goco N, Hemingway-Foday J, Moore J, Nolen TL, McClure EM, Koso-Thomas M, Miodovnik M, Silver R, Derman RJ, ASPIRIN Study Group (2020). Low-dose aspirin for the prevention of preterm delivery in nulliparous women with a singleton pregnancy (ASPIRIN): a randomized, double-blind, placebo-controlled trial. Lancet.

[28] Usman F, Imam A, Farouk ZL, Dayyabu AL (2019). Newborn Mortality in Sub-Saharan Africa: Why is Perinatal Asphyxia Still a Major Cause?. Ann Glob Health.

[29] Lee AC, Kozuki N, Blencowe H (2013). Intrapartum-related neonatal encephalopathy incidence and impairment at regional and global levels for 2010 with trends from 1990. Pediatr Res.

[30] Wall SN, Lee AC, Niermeyer S, et al. Neonatal resuscitation in low-resource settings: what, who, and how to overcome challenges to scale up?. Int J Gynaecol Obstet. 2009;107(Suppl 1):S47–64. 10.1016/j.ijgo.2009.07.013. 10.1016/j.ijgo.2009.07.013 PMC2875104 19815203

[31] Ersdal HL, Mduma E, Svensen E, Perlman J (2012). Birth asphyxia: a major cause of early neonatal mortality in a Tanzanian rural hospital. Pediatrics..

[32] Gupta N, Castillo-Laborde C, Landry MD (2011). Health-related rehabilitation services: assessing the global supply of and need for human resources. BMC Health Serv Res.

